# Supplementary material for: Humoral Immune Response Following COVID-19 Vaccination in Multifocal Motor Neuropathy and Chronic Inflammatory Demyelinating Polyneuropathy
Source: Vaccines (Basel). 2025 Aug 26;13(9):902. doi: 10.3390/vaccines13090902 (PMC12474186; doi:10.3390/vaccines13090902)
Supplement: Supplementary file 1 [file vaccines-13-00902-s001.zip › vaccines-3778008-supplementary.pdf]

Supplementary Table S1. Distribution of vaccine types across doses.

Vaccine Type	1st Dose	2nd Dose	3rd Dose	4th Dose	5th Dose	6th Dose
Pfizer BioNTech BNT162b2	317	335	330	31	NA	
Pfizer (Omicron BA.1)				100	NA	
Pfizer (Omicron BA.4-5)				172	9	
Pfizer BioNTech Comirnaty Omikron XBB.1.5				NA	18	NA
Pfizer BioNTech Comirnaty JN. 1						8
Moderna / Spikevax	NA	NA	5	NA		
Spikevax JN1						6
Astra Zeneca ChAdOx1	21					

The numbers represent the count of healthy controls and patients who received the vaccine. If fewer than five individuals were included, 'NA' is indicated.
Grey-shaded cells indicate when the vaccine was not available for administration.

Supplementary Table S2.

Table S2A. COVID-19 infections per visit detected by Nucleocapsid antibodies in patients with MMN, CIDP, and controls during the study.

Visit	Baseline	21 days	3 months	6 months	12 months	18 months	24 months
Controls,<br>No. infected (%)	21 / 297<br>(7.1 %)	14 / 221<br>(6.3 %)	9 /145<br>(6.2 %)	10 / 188<br>(5.3 %)	32 / 213<br>(15 %)	113 / 167<br>(67.7 %)	113 / 154<br>(73.4 %)
Patients with MMN and CIDP,<br>No. infected (%)	2 / 32<br>(6.2 %)	2 / 25<br>(8.9 %)	2 / 29<br>(6.9 %)	14 / 29<br>(48.3 %)	14 / 28<br>(50.0 %)	16 / 24<br>(66.7 %)	16 / 27<br>(59.3 %)

No, Number of; MMN, Multifocal Motor Neuropathy; CIDP, Chronic Inflammatory Demyelinating Polyneuropathy.

Table S2B. Differences in SARS-CoV-2 anti-RBD IgG levels across visits between controls and patients with MMN and CIDP adjusted for current COVID-19 infection detected by Nucleocapsid antibodies at time of sampling.

Visit	Mean difference, %	Estimate rate ratio	95% CI	p-value
Baseline	- 29 %	0.71	(0.32 – 1.56)	0.389
21 days	+ 29 %	1.29	(0.65 – 2.57)	0.461
3 months	+ 31 %	1.31	(0.84 – 2.05)	0.234
6 months	+ 223 %	3.23	(2.06 – 5.05)	<0.001*
12 months	+ 134 %	2.34	(1.50 – 3.63)	<0.001*
18 months	+ 77 %	1.77	(1.12 – 2.80)	0.015*
24 months	+ 106 %	2.06	(1.39 – 3.05)	<0.001*

Mean difference and the estimated rate ratio are reported with patients as the reference group. A mean difference of 77% means that the controls had 77% higher SARS-CoV-2 anti-Receptor-binding domain Immunoglobulin G levels compared to the patient group. Asterisks indicate statistical significance ($p < 0.05$).
Abbrevations: 95% CI, 95% Confidence Interval; MMN, Multifocal Motor Neuropathy; CIDP, Chronic Inflammatory Demyelinating Polyneuropathy.

Table S2C. Patients with MMN and CIDP and controls with reinfections (≥ 2 COVID-19 infections) detected by Nucleocapsid antibodies throughout the study.

	Individuals with reinfections* / Total no. participants	Percentages
Controls	99 / 306	32.4 %
Patients with MMN and CIDP	17 / 34	50.0 %

*Reinfections was defined as detected Nucleocapsid antibodies at 2 or more visits throughout the study.
Abbrevations: MMN, Multifocal Motor Neuropathy; CIDP, Chronic Inflammatory Demyelinating Polyneuropathy.
